# Elucidating the role of negative parenting in the genetic *v.* environmental influences on adult psychopathic traits

**DOI:** 10.1017/S0033291721002269

**Published:** 2023-02

**Authors:** Hailey L. Dotterer, Alexandra Y. Vazquez, Luke W. Hyde, Craig S. Neumann, Pekka Santtila, Patrizia Pezzoli, Ada Johansson, S. Alexandra Burt

**Affiliations:** 1 Department of Psychology, University of Michigan, Ann Arbor, MI, USA; 2 Department of Psychology, Michigan State University, East Lansing, MI, USA; 3 Survey Research Center of the Institute for Social Research, University of Michigan, Ann Arbor, MI, USA; 4 Department of Psychology, University of North Texas, Denton, TX, USA; 5 NYU-ECNU Institute for Social Development, NYU Shanghai, Shanghai, China; 6 Institute of Mental Health Research, University of Ottawa, Ontario, CA, Canada; 7 Faculty of Medicine, University of Ottawa, Ottawa, Canada; 8 Faculty of Arts, Psychology, and Theology, Åbo Akademi University, Turku, Finland

**Keywords:** Behavioral genetics, bivariate ACE model, parenting, psychopathy

## Abstract

**Background:**

Psychopathic traits involve interpersonal manipulation, callous affect, erratic lifestyle, and antisocial behavior. Though adult psychopathic traits emerge from both genetic and environmental risk, no studies have examined etiologic associations between adult psychopathic traits and experiences of parenting in childhood, or the extent to which parenting practices may impact the heritability of adult psychopathic traits using a genetically-informed design.

**Methods:**

In total, 1842 adult twins from the community reported their current psychopathic traits and experiences of negative parenting during childhood. We fit bivariate genetic models to the data, decomposing the variance within, and the covariance between, psychopathic traits and perceived negative parenting into their genetic and environmental components. We then fit a genotype × environment interaction model to evaluate whether negative parenting moderated the etiology of psychopathic traits.

**Results:**

Psychopathic traits were moderately heritable with substantial non-shared environmental influences. There were significant associations between perceived negative parenting and three of four psychopathy facets (interpersonal manipulation, erratic lifestyle, antisocial tendencies, but not callous affect). These associations were attributable to a common non-shared environmental pathway and not to overlapping genetic effects. Additionally, we found that primarily shared environmental influences were *stronger* on psychopathic traits for individuals with a history of greater negative parenting.

**Conclusions:**

Utilizing a genetically-informed design, we found that both genetic and non-shared environmental factors contribute to the emergence of psychopathic traits. Moreover, perceptions of negative parenting emerged as a clear environmental influence on the development of interpersonal, lifestyle, and antisocial features of psychopathy.

Psychopathic traits, including superficial charm, callousness, irresponsibility, and poor impulse control, are strongly predictive of violence, criminality, and recidivism (Hare & Neumann, 2008; Olver et al., 2018; Reidy et al., 2015). Though historically thought to be a highly genetic/heritable disorder (Skeem, Polaschek, Patrick, & Lilienfeld, 2011), researchers have moved to a more complex and nuanced understanding of the etiology of psychopathy, emphasizing the role of both genetic and environmental influences (Viding & McCrory, 2018). Indeed, although psychopathic traits are moderately heritable (e.g. A = 24–67%; Rhee & Waldman, 2002), there is extensive evidence that they are also impacted by environmental factors (Tuvblad & Baker, 2011; Tuvblad, Fanti, Andershed, Colins, & Larsson, 2017; Viding & McCrory, 2012; Waldman, Rhee, LoParo, & Park, 2018).

One potentially important environmental influence in the development of psychopathy is harsh parenting. Harsh parenting has been robustly linked to broader antisocial behavior in youth (Loeber, Burke, & Pardini, 2009; Moffitt, 2018; Waller, Gardner, & Hyde, 2013) and adults (Farrington, 2000; Loeber & Hay, 1997; Moffitt, 2018). Moreover, accumulating research in youth shows that harsh parenting is a robust predictor of callous-unemotional (CU) traits (Waller et al., 2013). CU traits are a downward extension of interpersonal and affective components of psychopathy (Salekin, 2017) and are a risk factor for adult psychopathy (Lynam, Caspi, Moffitt, Loeber, & Stouthamer-Loeber, 2007). Thus, although research on parental influences on CU traits can inform our understanding of the etiology of psychopathy, research is needed to confirm the role that parents play in the development of the broader manifestation of psychopathy in adulthood.

Consistent with youth studies of CU traits, emerging research suggests that parenting practices are correlated with psychopathic traits in adult offspring (Eisenbarth, Krammer, Edwards, Kiehl, & Neumann, 2018; Piquero et al., 2012). However, these findings may reflect a genotype–environment correlation (rGE). As parenting itself is moderately heritable (Klahr & Burt, 2014), parents with genetic risk for psychopathic traits may be more likely to utilize harsh parenting practices (passive rGE) (Beaver et al., 2014; Cox, Kopkin, Rankin, Tomeny, & Coffey, 2018). Alternately, children with genetic risk for psychopathic traits may evoke harsher parenting reactions (evocative rGE) (Hawes, Dadds, Frost, & Hasking, 2011). Research is thus needed to determine the extent to which associations between parenting and subsequent psychopathic traits are genetic *v.* environmental in origin.

Several studies of youth have used genetically informed designs to elucidate the origins of the association between parenting and CU traits. For example, positive parenting has been shown to predict CU traits using an adoption design that eliminates passive rGE (Hyde et al., 2016). Parental harshness and warmth were also associated with CU traits using a twin difference design that indexes non-shared environmental influences (Waller, Hyde, Klump, & Burt, 2018). In contrast, a longitudinal twin difference study found that differences in negative parental discipline (age 7) did *not* predict differences in CU traits (age 12), when accounting for earlier levels of CU traits (Viding, Fontaine, Oliver, & Plomin, 2009). Moreover, other studies have suggested that parenting may moderate the etiology of CU traits. For example, Hyde et al. (2016) found that positive parenting by adoptive mothers buffered the effects of genetic risk for CU traits inherited from biological mothers. Similarly, a recent twin study by Henry et al. (2018) found that positive parenting moderated the heritability of CU traits, such that CU traits were less heritable in the presence of positive parenting (i.e. positive parenting buffered against genetic risk).

Collectively, these studies suggest that parenting may be an environmental influence on CU traits and may moderate the genetic influences on the development of CU traits. Critically, however, most of the prior genetically informed studies have examined the impact of positive parenting practices on CU traits (e.g. Henry et al., 2018; Hyde et al., 2016). This is an important distinction since positive and negative parenting are not mirror images of one another (i.e. positive parenting is not the absence of harsh parenting but the presence of warmth and praise, etc.). Consistent with this, extant literature suggests that associations with CU traits may differ for positive *v.* harsh parenting (Viding et al., 2009; Waller et al., 2018). Further research is thus needed to determine the impact of negative parenting practices on CU traits, particularly potential genotype × environment interactions.

What's more, extant literature has largely focused on youth CU traits, leaving open the major question of whether any of these findings extend into adulthood and to a broader and more complex manifestation of psychopathy (Neumann, Hare, & Pardini, 2015). This is a key issue since genetic and environmental influences on psychopathic traits and antisocial behavior appear to change over time (Ferguson, 2010; Rhee & Waldman, 2002). As such, etiologic correlations between parenting and psychopathic traits may differ in adults with psychopathic traits as compared to youth with CU traits. However, no studies have examined the extent to which associations between parenting received in childhood and adult psychopathic traits are explained by genetic *v.* environmental influences, or whether parenting moderates heritability estimates of adult psychopathic traits.

Finally, etiologic associations between early experiences of parenting and adult psychopathic traits may also differ across the different facets of psychopathy. Psychopathy has commonly been parsed into four related, but unique, facets (interpersonal manipulation, callous affect, erratic lifestyle, and antisocial tendencies). These facets are characterized by distinct external correlates, suggesting possibly distinct etiologies (Blonigen, Hicks, Krueger, Patrick, & Iacono, 2006; Carré, Hyde, Neumann, Viding, & Hariri, 2013; Hare & Neumann, 2005; Hoppenbrouwers, Neumann, Lewis, & Johansson, 2015; Witt, Donnellan, & Blonigen, 2009). For example, previous findings suggest that an interpersonal-affective factor (combining interpersonal manipulation and callous-affect facets) may be equally influenced by genetic and environmental factors, whereas an impulsive-antisocial factor (combining erratic lifestyle and antisocial tendencies facets) may have to be more strongly influenced by the environment than by genetics (Brook et al., 2010). Previous work has also found differential phenotypic correlations between dimensions of parenting and the different facets of psychopathy (e.g. Vachon, Lynam, Loeber, & Stouthamer-Loeber, 2012), highlighting the need to examine these questions at the facet level.

## Current study

The current study addresses these gaps in the literature by the etiology of the association between psychopathic traits in adulthood and experiences of negative parenting in childhood. We first examined etiologic associations among each of the psychopathy facets (interpersonal manipulation, callous affect, erratic lifestyle, and antisocial tendencies) and retrospective reports of negative parenting using bivariate ACE modeling. We predicted that there would be a significant overlap between non-shared environmental contributions to negative parenting and those for psychopathic traits. We then examined whether negative parenting moderated genetic and environmental influences on psychopathic traits. In doing so, we were guided by bioecological models that suggest that environmental influences can take on a larger role in harsh environments because there is more ‘push’ in these environments toward maladaptive outcomes, an important model for characterizing G × E in antisocial behavior (Burt, 2015; Raine, 2002). That is, psychopathic traits may be more heritable in lower risk environments (i.e. no exposure to harsh parenting), but less heritable in higher risk environments (i.e. exposure to harsh parenting), potentially because there is more environmental risk in adverse contexts. Thus, we predicted that the genetic influence on psychopathic traits would be *lower* and environmental components of psychopathic traits would be *higher* for individuals who reported receiving more negative parenting.

## Methods

### Participants

The present study examined a population-based sample of Finnish twins who participated in the Genetics of Sexuality and Aggression study (Johansson et al., 2013). Participants were recruited through the Central Population Registry of Finland and were 18–49 years of age [mean of 37.5 years (s.d. = 3.06)] at the time of data collection (Johansson et al., 2013). The current sample consisted of 1842 adults in 921 twin pairs from the first wave of data collection: 91 male MZ pairs, 247 female MZ pairs, 110 male DZ pairs, 271 female DZ pairs, and 202 opposite-sex DZ pairs. Zygosity was determined with questionnaire items completed by the twins, a method which is 95% accurate when compared with blood typing analyses (Sarna, Kaprio, Sistonen, & Koskenvuo, 1978). All procedures contributing to this work comply with the ethical standards of the Board for Research Ethics at Åbo Akademi University and with the Helsinki Declaration of 1975, as revised in 2008.

### Measures

#### Self-reported psychopathic traits

Psychopathic traits were assessed using the 19-item experimental version of the Self-Report Psychopathy (SRP-E), a self-report measure of psychopathy derived from the Psychopathy Checklist-Revised and other versions of the Self-Report of Psychopathy (Neumann, Schmitt, Carter, Embley, & Hare, 2012; Paulhus, Neumann, & Hare, 2009). Items are grouped into four facets: interpersonal manipulation (e.g. ‘I think I can beat a lie detector’), affective callousness (e.g. ‘I never feel guilty over hurting others’), erratic lifestyle (e.g. ‘I've often done dangerous things just for the thrill of it’), and antisocial tendencies (e.g. ‘I have broken into a building or vehicle in order to steal something or vandalize’) (Neumann et al., 2012; Paulhus et al., 2009). Though these facets show low to acceptable internal consistency (ranging from 0.48–0.77), they loaded onto four factors with acceptable model fit using confirmatory factor analysis in Mplus vs 8.3 (Muthén & Muthén, 2019) (see online Supplementary Fig. S1).

#### Retrospective reports of parenting

Recalled negative parenting was assessed using a total sum score of the Measure of Parenting Style (*α* = 0.94), a 15-item self-report questionnaire (MOPS; Parker et al., 1997) used to assess participant perceptions of their parents' behaviors and attitudes until the participants were 16 years of age. Because twin-reported negative maternal and paternal parenting were highly correlated (*r* = 0.57; Table 1), we created composite perceptions of negative parenting via averages. Results were also run separately for maternal *v.* paternal parenting, and did not alter the conclusions (see Supplemental Materials).

**Table 1.** Descriptive statistics and phenotypic correlations

		Monozygotic twins				Dizygotic twins				
Construct	Mean	s.d.	Minimum	Maximum	*N*	Mean	s.d.	Minimum	Maximum	*N*
Perceived negative parenting	1.73	0.48	1.00	3.43	628	1.72	0.44	1.00	3.47	1073
Callous affect	2.36	0.57	1.00	4.60	610	2.39	0.55	1.00	4.40	993
Antisocial tendencies	1.27	0.56	1.00	5.00	610	1.29	0.59	1.00	5.00	993
Interpersonal manipulations	1.75	0.60	1.00	4.75	610	1.74	0.57	1.00	4.50	993
Erratic lifestyle	1.97	0.65	1.00	4.33	610	2.01	0.62	1.00	4.67	993

Phenotypic correlations							
Phenotype	Maternal perceived negative parenting	Parental perceived negative parenting	Composite perceived negative parenting	Callous affect	Antisocial tendencies	Interpersonal manipulation	Erratic lifestyle
Perceived negative maternal parenting	1	0.57* (0.54, 0.60)	0.88* (0.86, 0.89)	0.04 (−0.01, 0.09)	0.17* (0.12, 0.22)	0.14* (0.09, 0.19)	0.15* (0.10, 0.19)
Perceived negative parental parenting	–	1	0.90* (0.89, 0.91)	0.05 (0, 0.10)	0.23* (0.18, 0.28)	0.17* (0.12, 0.22)	0.19* (0.14, 0.24)
Composite perceived negative parenting	–	–	1	0.04 (−0.01, 0.09)	0.23* (0.18, 0.28)	0.18* (0.13, 0.23)	0.20* (0.15, 0.24)

*Note.* *p ⩽ 0.05. s.d. and *N* refer to standard deviation and sample size, respectively. Descriptives for the psychopathy facet scores are based on unstandardized values.

### Statistical analyses

Classical twin studies leverage the difference in the proportion of genes shared between monozygotic or MZ twins (who share 100% of their genes) and dizygotic or DZ twins (who share an average of 50% of their segregating genes) to estimate the relative contributions of genetic (A), shared environmental (C), and non-shared environmental influences to the variance within observed behaviors or characteristics (phenotypes). More information on twin studies is provided elsewhere (Neale & Cardon, 1992).

We first fitted bivariate ACE models to characterize the associations between perceived parenting and the psychopathy facets. This model parses the phenotypic variance of perceived negative parenting and the phenotypic covariance between perceived parenting and a given psychopathy facet into that which is due to genetic, shared environmental, and non-shared environmental factors. The genetic and environmental covariances were then standardized on their respective variances to produce genetic and environmental correlations. These statistics reveal the extent to which a specific effect (e.g. the genetic effect) on perceptions of negative parenting received is correlated with the same effect on each of three psychopathy facets. The bivariate model therefore enabled us to offer focused conclusions on the etiology of overlap between perceived parenting and psychopathic traits.

We also evaluated whether perceived negative parenting moderated the etiology of the psychopathy facets using the extended univariate genotype-by-environment interaction (G × E) model (van der Sluis, Posthuma, & Dolan, 2012), an extension of the univariate G × E model (Purcell, 2002). Perceived negative parenting was first entered in a means model of each twin's psychopathy facet score. Linear moderation was then modeled on the residual psychopathy facet variance (i.e. that which does not overlap with perceived negative parenting), thereby controlling for any genotype–environment correlations. For each phenotype, we fitted a no moderation model, a full ACE moderation model, and a series of nested models in which small or non-significant moderators were constrained to 0. A variety of fit indices (detailed below) were then utilized to determine the best-fitting model. As recommended in prior work (van der Sluis et al., 2012), we also fitted bivariate G × E models as an important clarification of our univariate G × E results (Purcell, 2002). Bivariate G × E models model the relationship between the moderator and the outcome using a Cholesky framework to distinguish between moderation of the covariance path and moderation of the residual path, thereby further clarifying the precise nature of the moderation.

Consistent with prior work (Burt, Clark, Pearson, Klump, & Neiderhiser, 2020; Burt, Pearson, Carroll, Klump, & Neiderhiser, 2020; Burt, Wildey, & Klump, 2015; Klump, Perkins, Burt, McGue, & Iacono, 2007), the moderator was floored at zero and divided by its highest value to have a range of 0–1 prior to analysis, an approach that facilitates the interpretation of the unstandardized model-fitting estimates. We statistically controlled for twin sex and age effects in both the bivariate and G × E biometric analyses via standard regression techniques (McGue & Bouchard, 1984).

Mplus version 8.3 (Muthén & Muthén, 2019) was used to perform the model-fitting analyses. Because of the small amount of missing data, we used Full-Information Maximum-Likelihood raw data techniques, which produce less biased and more efficient estimates than pairwise or listwise deletion in the face of missing data (Yuan & Bentler, 2000). When fitting models to raw data, variances, covariances, and means are first freely estimated to get a baseline index of fit (minus twice the log-likelihood; −2lnL). The −2lnL under this unrestricted baseline model is then compared with −2lnL under more restrictive biometric models. This comparison provides a likelihood-ratio χ^2^ test of goodness of fit for the model, which is then converted to the Akaike's information criterion (Akaike, 1987; AIC = χ^2^–2df). The AIC measures model fit relative to parsimony; better fitting models have more negative values. Additionally, the Root Mean Square Error of Approximation (RMSEA) and Comparative Fit Index (CFI) were utilized as additional indices of absolute and relative fit, respectively. Models with an RMSEA ⩽0.05 and CFI ⩾0.90 are judged to have a good fit (Cheung & Rensvold, 2002; Yuan & Bentler, 2000). Confidence intervals were derived using non-parametric bootstrapping, which provides reliable confidence intervals for assessing parameter estimate precision under a variety of complex data conditions without concerns for violating the typical assumptions of structural equation models (Falk, 2018). Significance was then determined via 95% confidence intervals that do not overlap with zero.

## Results

### Descriptive statistics and correlations

See Table 1 for descriptive statistics. Negative perceived parenting was associated with all facets of psychopathy (*r*s = 0.17–0.23), except for callous affective (*r*s = 0.04–0.05) (Table 1). Intraclass and cross-twin cross-trait correlations are presented in the online Supplementary Table 1. The MZ intraclass correlations were greater than twice the DZ intraclass correlations for all facets of psychopathy, indicating a high degree of heritability for these traits. The MZ intraclass correlation for perceived negative parenting was slightly less than twice the DZ intraclass correlation, implying the presence of clear genetic influences but also the presence of shared environmental influences. Additionally, nearly all cross-twin cross-trait correlations among the psychopathy facets were larger in MZ twins compared to DZ twins, suggesting that the co-occurrence of psychopathy facets is attributable to genetic influences. In contrast, cross-twin cross-trait correlations between each psychopathy facet and negative perceived parenting were equivalent, suggesting that environmental influences play a key role in the co-occurrence of perceived negative parenting and psychopathic traits, with minimal genetic overlap.

### Univariate and bivariate analyses

The ACE model fit the data well for each of the four sets of models (Table 2). Univariate ACE estimates (Table 3) revealed that perceived negative parenting was significantly influenced by genetics (42%) and shared (16%) and non-shared environment (42%). Because our measure of parenting indexes perceptions of parenting received as a child, these genetic effects are thought to reflect a genotype–environment correlational process in which people shape the parenting that they receive in part as a function of their genetically-influenced characteristics (Klahr & Burt, 2014; Pezzoli, Antfolk, Hatoum, & Santtila, 2019). The univariate ACE estimates for psychopathic traits also consistently demonstrated significant, albeit moderate, heritability (28–36%, *p* < 0.05). By contrast, non-shared environmental influences were prominent (42–68%, *p* < 0.05) and shared-environmental influences were trivial (0–7%).

**Table 2.** Model fit statistics

Model	*N* (MZ/DZ)	Log likelihood (H0/H1 OR H0) (df)	AIC	BIC	SBIC	χ^2^(df)	RMSEA	CFI
Callous affect univariate	328/563	−2243.89/−2239.38	4495.75	4514.92	4502.22	8.991(6)	0.033	0.948
Perceived negative parenting/antisocial tendencies bivariate	337/583	−4498.45/−4482.80	9018.91	9071.97	9037.04	31.30 (17)	0.04	0.96
Perceived negative parenting/interpersonal manipulation bivariate	337/583	−4514.26/−4504.53	9050.53	9103.59	9068.66	19.47 (17)	0.02	0.99
Perceived negative parenting/erratic lifestyle bivariate	337/583	−4515.48/−4507.09	9052.96	9106.03	9071.09	16.78 (17)	0	1
Callous affect univariate G × E								
ACE	299/506	−1932.76 (12)	3889.53	3945.82	3907.71	–	–	–
C	299/506	−1933.09 (10)	3886.18	3933.09	3901.33	–	–	–
No moderation	299/506	−1934.91 (9)	3887.82	3930.04	3901.46	–	–	–
Antisocial tendencies univariate G × E								
ACE	299/506	−1857.64 (12)	3739.27	3795.56	3757.45	–	–	–
CE	299/506	−1857.73 (11)	3737.47	3789.07	3754.14	–	–	–
No moderation	299/506	−1874.90 (9)	3767.73	3810.01	3781.43	–	–	–
Interpersonal manipulation univariate G × E								
ACE	299/506	−1906.03 (12)	3836.06	3892.35	3854.24	–	–	–
C	299/506	−1907.02 (10)	3834.04	3880.95	3849.19	–	–	–
No moderation	299/506	−1911.76 (9)	3841.53	3883.75	3855.17	–	–	–
Erratic lifestyle univariate G × E								
ACE	299/506	−1909.80 (12)	3843.59	3899.88	3861.78	–	–	–
C	299/506	−1910.65 (10)	3841.31	3888.24	3856.46	–	–	–
No moderation	299/506	−1915.22 (9)	3848.44	3890.66	3862.08	–	–	–
Perceived negative parenting/antisocial tendencies bivariate G × E	299/506	−1312.42	2658.84	2738.59	2684.60			
Perceived negative parenting/interpersonal manipulation bivariate G × E	299/506	−1386.69	2807.37	2887.12	2833.13			
Perceived negative parenting/erratic lifestyle bivariate G × E	229/506	−1363.17	2760.34	2840.08	2786.10			

*Note.* MZ, DZ, AIC, BIC, SBIC, χ^2^(df), RMSEA, and CFI refer to monozygotic, dizygotic, Akaike's information criterion, Bayesian information criterion, sample-size adjusted Bayesian information criterion, χ^2^ (degrees of freedom), Root Mean Square Error of Approximation, and Comparative Fit Index, respectively.

**Table 3.** ACE estimates and correlations

Model	A	C	E	rA	rC	rE
Perceived negative parenting univariate	0.42* (0.22, 0.59)	0.16* (0.01, 0.33)	0.42* (0.36, 0.50)	–	–	–
Callous affect univariate	0.36* (0.26, 0.44)	0.00 (0, 0)	0.64* (0.56, 0.73)	–	–	–
Antisocial tendencies univariate	0.30* (0.06, 0.44)	0.07* (0.001, 0.24)	0.63* (0.53, 0.74)	–	–	–
Interpersonal manipulation univariate	0.30* (0.12, 0.41)	0.05 (0, 0.19)	0.65* (0.57, 0.74)	–	–	–
Erratic lifestyle univariate	0.28* (0.07, 0.39)	0.05 (0, 0.20)	0.68* (0.59, 0.77)	–	–	–
Perceived negative parenting/antisocial tendencies bivariate	–	–	–	0.004 (−0.91, 0.36)	1.00* (0.01, 1)	0.29* (0.19, 0.39)
Perceived negative parenting/interpersonal manipulation bivariate	–	–	–	−0.17 (−0.99, 0.19)	1.00 (−1, 1)	0.34* (0.23, 0.43)
Perceived negative parenting/erratic lifestyle bivariate	–	–	–	−0.09 (−1, 0.28)	1.00 (−1, 1)	0.31* (0.21, 0.40)

Note. Standardized ACE estimates are reported. Asterisks indicate that the estimate is significant at *p* ⩽ 0.05. rA, rC, and rE refer to additive genetic correlation, shared environment correlation, and non-shared environment correlation, respectively.

Genetic and environmental correlations between parenting and the three psychopathy facets are also reported in Table 3. As we cannot decompose non-significant phenotypic associations into their genetic and environmental components, we omitted callous affective from the bivariate analyses. Non-shared environmental correlations between psychopathic traits and perceived negative parenting were all significant, ranging from 0.29 to 0.34, indicating a moderate overlap in the non-shared environmental influences on psychopathic traits and perceived negative parenting. Conversely, additive genetic correlations between psychopathic traits and perceived negative parenting ranged from −0.17 to 0.004 and were not significant, suggesting that the genetic effects influencing these phenotypes are largely unique to each (i.e. associations between perceived negative parenting and psychopathic traits are not substantially genetic). Finally, shared environmental correlations between perceived negative parenting and psychopathic traits were estimated at 1.00 and were non-significant or marginally significant. Note that high, but non-significant, shared environmental correlations are relatively common when there is very little shared environmental variance within a trait (Klahr, Thomas, Hopwood, Klump, & Burt, 2013). Though these results theoretically indicate that the shared environmental influences on perceived negative parenting overlap entirely with those on psychopathic traits, they have little practical meaning because there were minimal shared environmental influences on psychopathic traits.

Online Supplementary Fig. S1 illustrates these findings via a Cholesky decomposition framework, presenting standardized genetic and environmental path estimates for the variation within psychopathic traits as well as between these traits and perceived negative parenting. As suggested by the genetic and non-shared environmental correlations presented above, the genetic covariance paths across psychopathic traits were not significant, while the non-shared environmental covariance paths were all significant. This analysis further supports the conclusion that non-shared environmental influences on psychopathic traits overlap to some degree with those on perceived negative parenting (with negligible genetic etiology).

### G × e interaction analyses

**Fig. 1.**
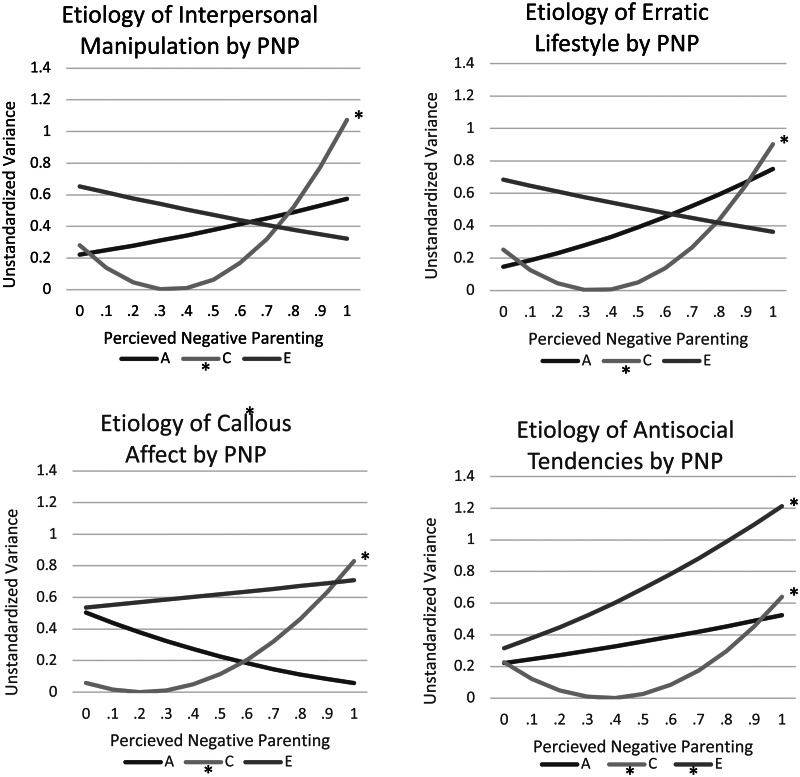
Etiologic moderation of the psychopathy facets by perceived negative parenting. Note. A, C, and E represent genetic, shared, and non-shared environmental influences, respectively. *Significant slope. These estimates index the absolute (unstandardized) changes in genetic and environmental variance in psychopathy by perceived negative parenting in a linear model. The specific path estimates are presented in Table 5. Of note, both significant and non-significant moderators are included in this figure to demonstrate the trend of all moderators. The slope for C is significant for all four models, whereas the slope for A is not significant in any, and the slope for E is only significant in the Antisocial Tendencies model (significance is indicated by asterisks to the right of the slope as well as in the ledger).

Next, we examined whether twins' perceptions of the negative parenting they received moderated the etiology of each of the four psychopathy facets (Tables 2 and 4; Fig. 1). Nested models fit best, and the etiology of all psychopathy facets varied as a function of perceived negative parenting. Across all psychopathy facets, absolute environmental contributions were more pronounced when participants reported greater levels of negative parenting. In particular, there were significantly greater shared-environmental contributions to all psychopathy facets and greater non-shared environmental contributions to antisocial tendencies when twins reported that they experienced particularly negative parenting in their childhoods. By contrast, absolute genetic contributions for all psychopathy facets were consistently equivalent across different levels of perceived negative parenting.

**Table 4.** Unstandardized path and moderator estimates for extended univariate genotype-by-environment models

	Path			Linear moderator		
	a	c	e	A_1_	C_1_	E_1_
Callous affect						
ACE	0.71* (0.43, 0.99)	−0.24 (−0.74, 0.26)	0.73* (0.63, 0.84)	−0.47 (−1.41, 0.47)	1.15* (0.31, 1.99)	0.11 (−0.19, 0.41)
C	0.58* (0.47, 0.69)	−0.42* (−0.76, −0.09)	0.76* (0.71, 0.82)	–	1.25* (0.43, 2.06)	–
Antisocial tendencies						
ACE	0.47* (0.05, 0.89)	−0.48* (−0.90, −0.05)	0.56* (0.43, 0.70)	0.25 (−0.80, 1.31)	1.28* (0.39, 2.17)	0.54* (0.14, 0.94)
CE	0.56* (0.39, 0.72)	−0.39* (−0.79, −0.001)	0.55* (0.43, 0.67)	–	1.28* (0.46, 2.11)	0.57* (0.23, 0.92)
Interpersonal manipulation						
ACE	0.47* (0.22, 0.72)	−0.53* (−0.79, −0.27)	0.81* (0.70, 0.92)	0.29 (−0.34, 0.97)	1.57* (0.93, 2.20)	−0.24 (−0.53, 0.06)
C	0.56* (0.47, 0.65)	−0.51* (−0.74, −0.29)	0.74* (0.69, 0.80)	–	1.58* (0.97, 2.19)	–
Erratic lifestyle						
ACE	0.39* (0.09, 0.68)	−0.50* (−0.79, −0.22)	0.83* (0.71, 0.95)	0.48 (−0.30, 1.27)	1.45* (0.68, 2.22)	−0.23 (−0.56, 0.11)
C	0.53* (0.43, 0.63)	−0.44* (−0.71, −0.18)	0.76* (0.71, 0.82)	–	1.51* (0.85, 2.17)	–

*Note.* A, C, and E (upper and lower case) respectively represent genetic, shared, and non-shared environmental parameters on psychopathy facets. Asterisks indicate that the estimate is significant at *p* ⩽ 0.05. Linear moderators (i.e. A_1_, C_1_, E_1_) were added to the paths using the following equation: Unstandardized Variance_Total_ = (a + A_1(PNP)_)^2^ + (c + C_1(PNP)_)^2^ + (e + E_1(PNP)_)^2^. The variance component estimates calculated this way are presented in the text. PNP, perceived negative parenting.

To clarify these results, we also fitted bivariate G × E models (Table 5; Purcell, 2002), as recommended by van der Sluis et al. (2012). For antisocial tendencies, the unique C and E moderators were estimated at 0.77 and 0.55 (*p* < 0.05), respectively, and the common C and E moderators were estimated at −0.61 and −0.23 (both ns), respectively. For interpersonal manipulation, the unique C moderator was estimated at 1.06 (*p* < 0.05), and the common C moderator was estimated at 0.04 (ns). For erratic lifestyle, the unique C moderator was estimated at 0.00, and the common C moderator was estimated at −1.24 (*p* < 0.05). These results indicate that the moderators for antisocial tendencies and interpersonal manipulation predominantly load onto the residual paths, a pattern of results that is interpreted as consistent with ‘true’ etiologic moderation. By contrast, the results for erratic lifestyle were less consistent with actual etiologic moderation of erratic lifestyle by parental negativity.

**Table 5.** Unstandardized path and moderator estimates for bivariate genotype-by-environment models

	Path			Linear moderator		
	a	c	e	A_1_	C_1_	E_1_
Perceived negative parenting/antisocial tendencies						
Shared	−0.25 (−0.52, 0.02)	0.52* (0.25, 0.80)	0..33* (0.18, 0.48)	0.57 (−0.07, 1.21)	−0.61 (−1.46, 0.24)	−0.23 (−0.65, 0.19)
Residual	0.22 (−0.15, 0.60)	−0.20 (−0.61, 0.21)	0.56* (0.44, 0.69)	0.66 (−0.20, 1.51)	0.77 (−0.25, 1.79)	0.55* (0.16, 0.94)
Perceived negative parenting/interpersonal manipulation						
Shared	−0.05 (−0.41, 0.31)	−0.10 (−0.52, 0.32)	0.22* (0.03, 0.40)	−0.10 (−0.95, 0.76)	0.04 (−1.10, 1.18)	−0.20 (−0.67, 0.27)
Residual	0.70* (0.39, 1.01)	−0.20 (−0.63, 0.24)	0.73* (0.63, 0.84)	−0.50 (−1.35, 0.36)	1.06* (0.07, 2.04)	0.11 (−0.19, 0.40)
Perceived negative parenting/erratic lifestyle						
Shared	0.19 (−0.09, 0.48)	0.56* (0.28, 0.84)	0.29* (0.13, 0.44)	−0.57 (−1.27, 0.14)	−1.24* (−2.17, −0.32)	−0.06 (−0.46, 0.34)
Residual	0.25 (−0.06, 0.56)	0.00 (−0.42, 0.42)	0.83* (0.71, 0.94)	0.79* (0.05, 1.53)	0.00 (−1.41, 1.41)	−0.23 (−0.56, 0.10)

*Note.* A, C, and E (upper and lower case) respectively represent genetic, shared, and non-shared environmental parameters on psychopathy facets. Asterisks indicate that the estimate is significant at *p* ⩽ 0.05. Linear moderators (i.e. A_1_, C_1_, E_1_) were added to the paths using the following equation: Unstandardized Variance_Total_ = (a + A_1(PNP)_)^2^ + (c + C_1(PNP)_)^2^ + (e + E_1(PNP)_)^2^. PNP, perceived negative parenting.

As a final set of robustness tests of our analyses, we sought to confirm that our interpretation did not vary when perceived negative maternal and paternal parenting were examined separately (see online Supplementary Tables 2 and 3), and to confirm that the etiology of the psychopathy facets did not vary with participant age (they did not; see online Supplementary Table 4). The pattern of environmental moderation reported above is fully replicated when examining parenting separately for mothers and fathers. Namely, pronounced and positively-signed shared environmental moderation by negative parenting was observed across all forms of psychopathy for both mothers and fathers. Similarly, we observed nearly identical levels of non-shared environmental moderation by negative parenting only for antisocial tendencies. That said, the patterns of genetic moderation differed across mothers and fathers relative to the composite, decreasing for two of the four forms of psychopathy when examining mothering and increasing for two of the four forms of psychopathy when examining fathering.

## Discussion

In a large population-based sample of adult twins, we found that psychopathic traits were moderately heritable with substantial non-shared environmental influences. We also observed significant associations between three of four facets of psychopathy in adulthood (interpersonal manipulation, erratic lifestyle, and antisocial tendencies) and twin perceptions of negative parenting received during their childhood. These associations were attributable to an overlap in non-shared environmental effects, whereas the genetic overlap was negligible. Moreover, G × E interaction analyses revealed that environmental influences on the interpersonal, lifestyle, and antisocial features of psychopathy were stronger for individuals who reported experiencing higher levels of perceived negative parenting during childhood. Thus, our findings highlight the role of both genetic and environmental factors in the etiology of psychopathic traits, while also highlighting the prominent role of parenting as an environmental influence on certain features of adult psychopathy (i.e. interpersonal, lifestyle, and antisocial).

The most basic contribution of this study was examining the relative contribution of genetic and environmental influences on psychopathy by extending this investigation to the facet level. Consistent with the existing literature on adult psychopathy (e.g. Benning, Patrick, Blonigen, Hicks, & Iacono, 2005; Hunt, Bornovalova, & Patrick, 2015; Larsson, Andershed, & Lichtenstein, 2006; Taylor, Loney, Bobadilla, Iacono, & McGue, 2003; Waldman et al., 2018), both genetic and non-shared environmental influences were significant. However, the current study found relatively genetic contributions (e.g. A = 28–36%) more consistent with lower bound estimates from prior studies of adult psychopathic traits (e.g. As = 24–67%) (Blonigen, Carlson, Krueger, & Patrick, 2003; Brook et al., 2010; Taylor et al., 2003; Tuvblad et al., 2019). It is unclear what may account for these differences across prior studies. These heritability estimates were also relatively similar across facets, suggesting that the etiology of each facet, at least as measured via twin analyses parsing broad heritable and environmental influences, is not divergent.

We also found that retrospective reports of negative parenting were correlated with higher levels of the interpersonal manipulation, erratic lifestyle, and antisocial tendencies largely for environmental reasons. This finding emphasizes that associations between recalled parenting and current features of psychopathy (i.e. interpersonal, lifestyle, and antisocial) are not due to gene–environment correlation, but are instead largely environmental in origin. Collectively, these studies are in contrast to lay theories of psychopathy as a ‘bad seed’ that is ‘born’ and not ‘made’ (Skeem et al., 2011). Instead, these results suggest that certain features of psychopathy (i.e. interpersonal, lifestyle, and antisocial), like aggression and antisocial behavior more broadly, have substantial, though moderate genetic influences, but with substantial non-shared environmental influences that include experiences such as negative parenting.

Somewhat surprisingly, however, perceived negative parenting was not associated with callous affect. This result conflicts with a larger body of previous work in youth that has linked negative parenting to CU traits, which most closely align with the callousness facet in adulthood (Waller et al., 2013). In the current study, the strongest associations between negative parenting and psychopathic facets were with the lifestyle and antisocial tendencies facets, which overlap the most with broader forms of antisocial behavior. Consistent with some work in youth (Waller et al., 2013, 2018), it may be that negative parenting is specifically important to antisocial behavior and antisocial behavioral components of psychopathy, whereas low positive parenting, such as parental warmth (which we did not measure here), may be key to understanding the interpersonal and affective features of psychopathy (Pasalich, Witkiewitz, McMahon, Pinderhughes, & Group, 2016; Waller & Hyde, 2017, 2018). On the other hand, the current study may instead be indexing the well-established environmental overlap between antisocial behavior and parenting, at least to the extent to which the facets of psychopathy measured here index antisocial behavior. Notably, though, negative parenting was also associated with the interpersonal facet, which has little overlap with antisocial behavior. At the least, it is important to note that these findings in adults do not necessarily translate directly to studies of youth, since studies of youth have focused mostly on CU traits and the closest facet measures here (callous affect) did not correlate with parenting.

Finally, similar to a recent study in youth focusing on CU traits and positive parenting (Henry et al., 2018), we found that perceived negative parenting moderated the etiology of three facets of psychopathy (i.e. interpersonal, lifestyle, antisocial). Specifically, for individuals reporting greater exposure to negative parenting, environmental influences were stronger. Based on the bivariate G × E results, negative parenting moderated the etiology of two facets specifically, interpersonal manipulation and antisocial tendencies, whereas there was weaker support for environmental moderation of the erratic lifestyle facet (i.e. moderator only loaded onto the shared pathway and is therefore less indicative of true etiologic moderation). Overall, this finding is consistent with the bioecological model of G × E (Bronfenbrenner & Ceci, 1994; Pennington et al., 2009), which posits that environmental influences take on a larger role in harsh environments because there is more ‘social push’ toward maladaptive outcomes in these environments (Raine, 2002). In this case, one interpretation is that parents who engaged in harsh social interactions with their children modeled and/or taught interpersonal approaches that include interpersonal manipulation, impulsive actions, and antisocial attitudes. Alternately, the children may have developed these attributes as a way of navigating the stress of harsh parenting. However, these results are also consistent with the differential susceptibility theory, which posits that some individuals are more sensitive to environment (both positive and negative) than others (Belsky & Pluess, 2009; Yan, Benner, Tucker-Drob, & Harden, 2017). In line with this hypothesis, we found greater genetic variance at the extreme end of environmental experience (i.e. high levels of perceived negative parenting) and minimal genetic variance for average environments. Further research will be important to better understand which theoretical framework most accurately reflects etiological mechanisms of psychopathic traits.

Notably, in a set of exploratory analyses examining perceived negative parenting by mothers *v.* fathers separately, the pattern of results largely replicated those reported for the parenting composite, pointing to potent environmental moderation of all four psychopathy facets regardless of whether parenting was assessed in regard to mothers or to fathers. That said, a few differences were observed. We specifically found that genetic influences on two of the four facets decreased as negative maternal parenting increased, the same as the directionality of associations when using the parent composite. However, we found that genetic influences on two of the four facets increased as perceived negative paternal parenting increased. It is unclear why these differed from each other or from the overall parenting composite. However, the inconsistency of the findings across operationalizations of negative parenting raises some doubt about their generalizability, and thus they should be interpreted with caution.

### Strengths and limitations

Using a large sample of twins, we parsed genetic and environmental influences on adult psychopathic traits (including interpersonal, affective, antisocial, and lifestyle features) and address significant gaps in the literature by conducting a bivariate analysis to examine the genetic and environmental overlap between perceived negative parenting in childhood and psychopathic traits in adulthood, as well as to examine negative parenting perceptions as a moderator of heritability estimates of psychopathic traits. These approaches have not been used in the study of adult psychopathy to date, and thus constitute an important contribution to the literature. Even so, the study had several important limitations. Adult participants retrospectively reported their experiences of parenting, which likely impacted the accuracy of their responses and could have been shaped by their current levels of psychopathy and/or to a third variable that influences both psychopathy and their response style (i.e. personality traits are linked to psychopathy and to how the participant reports on the parenting they received). This is potentially problematic since previous research has found that recall bias is correlated with the degree of psychopathology (Brewin, Andrews, & Gotlib, 1993). It is thus possible that the use of retrospective reports inflated environmental estimates, or genetic influences, in some way. Notably, however, the G × E models used here explicitly evaluated moderation only of that psychopathy variance that did not overlap with parenting. Because shared informant variance should act to artefactually increase correlations between parenting and psychopathy, we suspect that our G × E analyses may have largely circumvented the issue of shared informant effects. What's more, we note that retrospective reports of the family environment are at least moderately correlated with prospective reports (Bell & Bell, 2018), suggesting that they have some utility for the current study. Indeed, the heritability estimates of parenting in the current study were similar to those found in a meta-analysis (Klahr & Burt, 2014).

Building on this point, although the validity of self-report measures of psychopathy has been historically questioned given that core features of the construct (e.g. deceitfulness, manipulation), more recent research has not been able to find associations between psychopathic traits and response style (Ray et al., 2013). In short, it is not clear what effect our use of retrospective self-reports of parenting may have had on our results. Future work should seek to replicate these findings using prospectively collected, multi-informant data for both parenting and psychopathy.

Next, as we only measured negative parenting, we were not able to examine the potentially different associations that may have emerged with a measure of positive parenting, and cannot directly compare our results to the previous literature on positive parenting (Henry et al., 2018; Hyde et al., 2016). Finally, as is necessary to recruit large-scale twin samples, our sample was representative of the broader community and not enriched for those in forensic settings. Thus, our results may not be generalizable to clinical or offender samples with potentially more severe levels of psychopathic traits.

## Conclusion

In conclusion, we identified a common non-shared environmental pathway between perceived negative parenting and three facets of adult psychopathy (interpersonal, lifestyle, and antisocial, but not callous affect). Further, we found that perceived negative parenting moderated environmental influences on interpersonal, lifestyle, and antisocial features of psychopathy by increasing their importance to psychopathy when perceptions of negative parenting were high. Additionally, we expanded on previous work by demonstrating that the four facets of psychopathy were all moderately heritable with significant non-shared environmental influences. Overall, our results suggest that both genetics and experience are important for the development of psychopathy, and that parenting may be a specific malleable risk factor for the interpersonal, lifestyle, and antisocial features of adult psychopathy that can be targeted in preventative interventions. Moreover, this study, combined with other genetically informed studies, supports the causal environmental role of parenting in the etiology of some facets within the psychopathy construct.

## Supplementary material

For supplementary material accompanying this paper visit https://doi.org/10.1017/S0033291721002269. click here to view supplementary material
