# Supplementary material for: Elucidating the role of negative parenting in the genetic v. environmental influences on adult psychopathic traits
Source: Psychol Med. 2021 Aug 12;53(3):897–907. doi: 10.1017/S0033291721002269 (PMC9976022; doi:10.1017/S0033291721002269)
Supplement: Supplementary file 1 [file S0033291721002269sup001.docx]

**Supplemental Materials**

**Measures**

**Self-reported psychopathic traits.** Psychopathic traits were assessed using the 19-item experimental version of the Self-Report Psychopathy (SRP-E), a self-report measure of psychopathy shown to correlate highly with the Psychopathy Checklist-Revised and other versions of the Self-Report of Psychopathy (Neumann, Schmitt, Carter, Embley, & Hare, 2012; Paulhus, Neumann, & Hare, 2009). To create factor scores for each of the four facets, we used confirmatory factor analysis (CFA) in Mplus vs 8.3 (Muthén & Muthén, 2019) with maximum likelihood estimation with robust standard errors (to account for skew and zero-inflation) (Yuan & Bentler, 2000). The scale loadings of the four facets were moderate-to-high (range, β = .41 - .91, *p <* .001). Correlations among the four factors were all moderate-to-high (range, r = .25 - .87, *p* < .001). Model fit was evaluated based on alternative fit indices (Brown, 2014): root mean squared error of approximation (RMSEA) < .05, standardized root mean residual (SRMR) < .05, comparative fit index (CFI) > .95, and non-normed fit index (NNFI) > .95. Model fit of the four-factor model was acceptable (CFI = .94, TLI = .93, RMSEA = .04 [95% CI: .044 - .052], SRMR = .05; Supplemental Figure 1). Notably, the RMSEA of the null model (RMSEA = .11) was less than .158 ; thus, the CFI is likely inflated (Kenny, 2020). We extracted factor scores for each individual to be used in the rest of the analyses.

Supplemental Table 1. Intraclass, and Cross-Twin Cross-Trait Correlations

|  |  | Perceived Negative Parenting | Callous Affect | Antisocial Tendencies | Interpersonal Manipulation | Erratic Lifestyle | Perceived Negative Parenting | Callous Affect | Antisocial Tendencies | Interpersonal Manipulation | Erratic Lifestyle |
| --- | --- | --- | --- | --- | --- | --- | --- | --- | --- | --- | --- |
|  |  | A | A | A | A | A | B | B | B | B | B |
| Perceived Negative Parenting | A | — | .06  (-.06, .17) | .26*  (.15, .36) | .20*  (.09, .31) | .22*  (.11, .33) | .62*  (.54, .68) | -.04  (-.16, .08) | .05  (-.07, .17) | -.01  (-.13, .12) | .01  (-.11, .13) |
| Callous Affect | A | .06  (-.03, .14) | — | .36*  (.26, .45) | .71*  (.65, .76) | .52*  (.43, .59) | -.05  (-.16, .06) | .40*  (.29, .49) | .24*  (.13, .35) | .33*  (.22, .43) | .28*  (.16, .38) |
| Antisocial Tendencies | A | .24*  (.15, .32) | .33*  (.25, .40) | — | .73*  (.67, .77) | .71*  (.65, .76) | .19*  (.08, .30) | .24*  (.12, .35) | .41*  (.31, .50) | .30*  (.19, .41) | .29*  (.18, .40) |
| Interpersonal Manipulation | A | .20*  (.11, .28) | .73*  (.69, .77) | .65*  (.60, .70) | — | .95*  (.94, .96) | .06  (-.05, .17) | .36*  (.26, .46) | .34*  (.23, .44) | .40*  (.29, .49) | .37*  (.26, .47) |
| Erratic Lifestyle | A | .23*  (.14, .31) | .53*  (.47, .59) | .65*  (.60, .70) | .95*  (.94, .95) | — | .09  (-.02, .20) | .31*  (.20, .41) | .33*  (.22, .43) | .37*  (.26, .47) | .36*  (.26, .46) |
| Perceived Negative Parenting | B | .34*  (.26, .41) | .04  (-.05, .12) | .10*  (.02, .19) | .09*  (.01, .18) | .10  (.02, .19) | — | .02  (-.10, .14) | .28*  (.16, .38) | .17*  (.05, .28) | .16*  (.04, .28) |
| Callous Affect | B | -.02  (-.11, .08) | .15*  (.06, .24) | -.02  (-.12, .07) | .07  (-.02, .16) | .04  (-.06, .13) | .07*  (-.03, .16) | — | .36*  (.25, .45) | .78*  (.73, .82) | .62*  (.54, .69) |
| Antisocial Tendencies | B | .12*  (.02, .21) | .09  (0, .18) | .20*  (.10, .29) | .16*  (.06, .25) | .16*  (.06, .25) | .27*  (.17, .35) | .35*  (.27, .43) | — | .68*  (.61, .74) | .68*  (.61, .74) |
| Interpersonal Manipulation | B | .03  (-.07, .12) | .16*  (.06, .25) | .09  (-.01, .18) | .16*  (.06, .25) | .13*  (.04, .22) | .22*  (.13, .31) | .68*  (.62, .72) | .74*  (.69, .77) | — | .96*  (.94, .96) |
| Erratic Lifestyle | B | .05  (-.05, .14) | .15*  (.06, .24) | .11*  (.01, .20) | .17*  (.08, .26) | .15*  (.06, .24) | .25*  (.16, .34) | .47*  (.39, .53) | .73*  (.68, .77) | .95*  (.94, .95) | — |

*Note.* Asterisks indicate that the estimate is significant at *p* < .05. ‘A’ refers to twin A, while ‘B’ refers to twin B. MZ twin correlations are above the diagonal and DZ twin correlations are below the diagonal.

Supplemental Table 2. ACE Estimates and Correlations for Maternal/Paternal Negative Perceived Parenting

| **Model** | **Maternal Parenting** | | | **Paternal Parenting** | | |
| --- | --- | --- | --- | --- | --- | --- |
|  | **A** | **C** | **E** | **A** | **C** | **E** |
| Perceived Negative Parenting  Univariate | 0.57*  (.36, .65) | 0.03  (0, .21) | 0.40*  (.33, .47) | 0.33*  (.12, .54) | 0.25*  (.07, .42) | 0.43*  (.35, .51) |
| Callous Affect  Univariate | 0.36*  (.27, .45) | 0.00  (0, 0) | 0.64*  (.56, .73) | 0.36*  (.27, .44) | 0.00  (0, 0) | 0.64*  (.56, .73) |
| Antisocial Tendencies Univariate | 0.37*  (.07, .47) | 0.01  (0, .23) | 0.62*  (.52, .73) | 0.28*  (.08, .42) | 0.09*  (.01, .23) | 0.63*  (.53, .74) |
| Interpersonal Manipulation Univariate | 0.33*  (.10, .42) | 0.02  (0, .20) | 0.65*  (.57, .75) | 0.33*  (.14, .41) | 0.03  (0, .18) | 0.65*  (.57, .74) |
| Erratic Lifestyle Univariate  Univariate | 0.30*  (.06, .40) | 0.03  (0, .21) | 0.67*  (.59, .78) | 0.30*  (.08, .40) | 0.03  (0, .19) | 0.67*  (.59, .77) |
|  | **rA** | **rC** | **rE** | **rA** | **rC** | **rE** |
| Perceived Negative Parenting / Antisocial Tendencies Bivariate | 0.28  (-.02, .80) | -1.00  (-1, 1) | .20*  (.10, .30) | -.22  (-1, .24) | 1.00*  (.56, 1) | 0.28*  (.16, .39) |
| Perceived Negative Parenting / Interpersonal Manipulation Bivariate | 0.04  (-.43, .28) | 1.00  (-1, 1) | .27*  (.18, .36) | -0.20  (-1, .24) | 1.00  (-1, 1) | 0.31*  (.12, .41) |
| Perceived Negative Parenting / Erratic Lifestyle Bivariate | 0.07  (-.49, .33) | 1.00  (-1, 1) | .25*  (.15, .34) | -0.12  (-1, 1) | 1.00  (-1, 1) | 0.28*  (.17, .38) |

Note. Standardized ACE estimates are reported. Asterisks indicate that the estimate is significant at *p* < .05. rA, rC, and rE refer to additive genetic correlation, shared environment correlation, and non-shared environment correlation, respectively.

Supplemental Table 3. Unstandardized Path and Moderator Estimates for Extended Univariate Genotype-by-Environment Models –Maternal and Paternal Perceived Negative Parenting

|  | Maternal Parenting - Path | | | Paternal Parenting - Path | | |
| --- | --- | --- | --- | --- | --- | --- |
|  | a | c | e | a | c | e |
| Callous Affect | 0.75*  (.59, .90) | -0.13  (-.48, .21) | 0.73*  (.64, .82) | 0.58*  (.14, 1.21) | -0.46*  (-.90, -.02) | 0.75*  (.64, .86) |
| Antisocial Tendencies | 0.54*  (.17, .92) | -0.52  (-.92, -.11) | 0.65*  (.53, .76) | 0.47*  (.14, .81) | -0.43*  (-.80, -.05) | 0.57*  (.45, .70) |
| Interpersonal Manipulation | 0.72*  (.56, .88) | -0.28*  (-.54, -.27) | 0.75*  (.65, .84) | 0.35*  (.08, .62) | -0.53*  (-.79, -.27) | 0.84*  (.73, .95) |
| Erratic Lifestyle | 0.68*  (.47, .90) | -0.29  (-.61, .03) | 0.76*  (.66, .86) | 0.34*  (.11, .58) | -0.40*  (-.75, -.06) | 0.87*  (.75, .98) |
|  | Maternal Parenting - Linear Moderator | | | Paternal Parenting - Linear Moderator | | |
|  | A_1_ | C_1_ | E_1_ | A_1_ | C_1_ | E_1_ |
| Callous Affect | -0.77*  (-1.41, -.13) | 1.10*  (.43, 1.78) | 0.19  (-.07, .44) | -0.04  (-1.28, 1.21) | 1.43*  (.56, 2.29) | 0.04  (-.32, .40) |
| Antisocial Tendencies | 0.01  (-1.09, 1.11) | 1.71*  (.81, 2.62) | 0.40*  (.03, .77) | 0.30  (-.69, 1.29) | 1.34*  (.47, 2.22) | 0.58*  (.14, 1.02) |
| Interpersonal Manipulation | -0.78*  (-1.37, -.19) | 1.60*  (1.00, 2.21) | 0.05  (-.27, .37) | 0.70*  (.02, 1.38) | 1.30*  (.65, 1.95) | -0.36  (-.71, -.004) |
| Erratic Lifestyle | -0.73  (-1.63, .16) | 1.64*  (1.01, 2.27) | 0.05  (-.26, .36) | 0.70*  (.08, 1.33) | 1.01*  (.10, 1.92) | -0.36  (-0.75, .04) |

*Note.* A, C, and E (upper and lower case) respectively represent genetic, shared, and non-shared environmental parameters on psychopathy facets. Asterisks indicate that the estimate is significant at *p* < .05. Linear moderators (i.e., A1, C1, E1) were added to the paths using the following equation: Unstandardized VarianceTotal = (*a* + *A*1(PNP))^2^ + (*c* + *C*1(PNP))^2^ + (*e* + *E*1(PNP))^2^. PNP = perceived negative parenting.

Supplemental Table 4. GxE Age Moderator Model Fit Statistics

| **Model** | ***N* (MZ/DZ)** | **Log Likelihood (H0)** | **AIC** | **BIC** | **SBIC** |
| --- | --- | --- | --- | --- | --- |
| Callous Affect | | | | | |
| ACE | 289/467 | -1950.46 | 3916.91 | 3953.94 | 3928.53 |
| No Moderation | 289/467 | -1952.61 | 3915.22 | 3938.36 | 3922.48 |
| Antisocial Tendencies | | | | | |
| ACE | 289/467 | -1906.60 | 3829.20 | 3866.22 | 3840.82 |
| No Moderation | 289/467 | -1907.88 | 3825.76 | 3848.90 | 3833.02 |
| Interpersonal Manipulation | | | | | |
| ACE | 289/467 | -1940.06 | 3896.12 | 3933.14 | 3907.74 |
| No Moderation | 289/467 | -1940.22 | 3890.45 | 3913.59 | 3897.71 |
| Erratic Lifestyle | | | | | |
| ACE | 289/467 | -1926.39 | 3868.77 | 3905.80 | 3880.40 |
| No Moderation | 289/467 | -1927.88 | 3865.76 | 3888.90 | 3873.02 |

*Note.* MZ, DZ, AIC, BIC, and SBIC refer to Monozygotic, Dizygotic, Akaike’s Information Criterion, Bayesian Information Criterion, and Sample-Size Adjusted Bayesian Information Criterion, respectively.

**
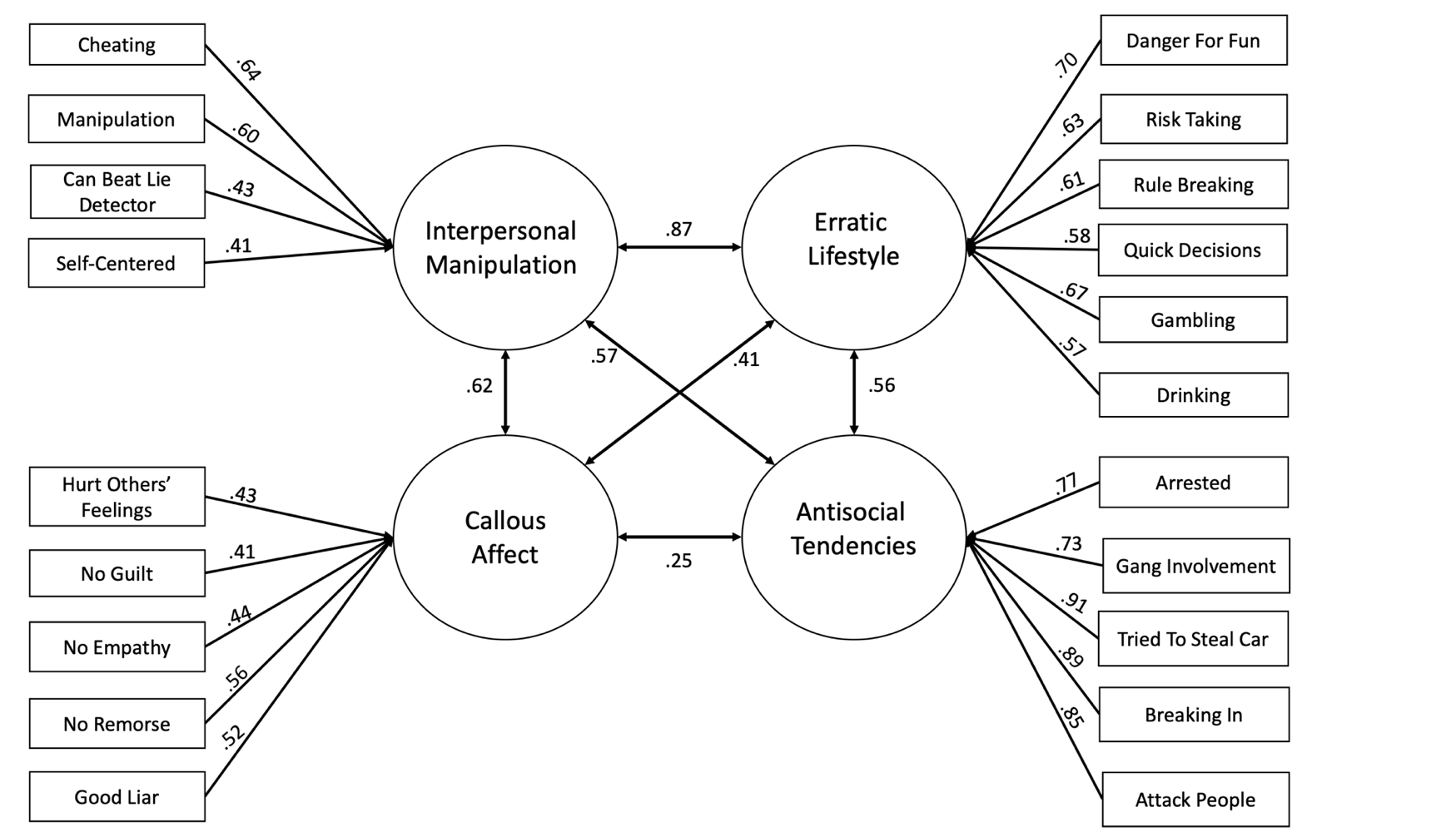
**

Supplemental Figure 1. Four-factor experimental Self-Report Psychopathy scale (SRP-E) model. Chi-Square Test of Model Fit: *x^2^* = 763.39, *df* = 164, *p* = .00. Model fit: CFI = .94, TLI = .93, RMSEA = .04 [95% CI: .044 - .052], SRMR = .05. Confirmatory factor analysis was performed using maximum likelihood estimation with robust standard errors in Mplus (which can accommodate skewness) to generate a normally distributed factor score to be used in analyses (Muthén & Muthén, 2019; Yuan & Bentler, 2000). All loadings and correlations were significant at *p* < .001.

Supplemental Figure 2. Standardized genetic and environmental bivariate path estimates. Note. Path estimates are presented, those with three estimates represent estimates for Antisocial Tendencies/Interpersonal Manipulation/Erratic Lifestyle in that order. Asterisks indicate that the estimate is significant at *p* < .05.


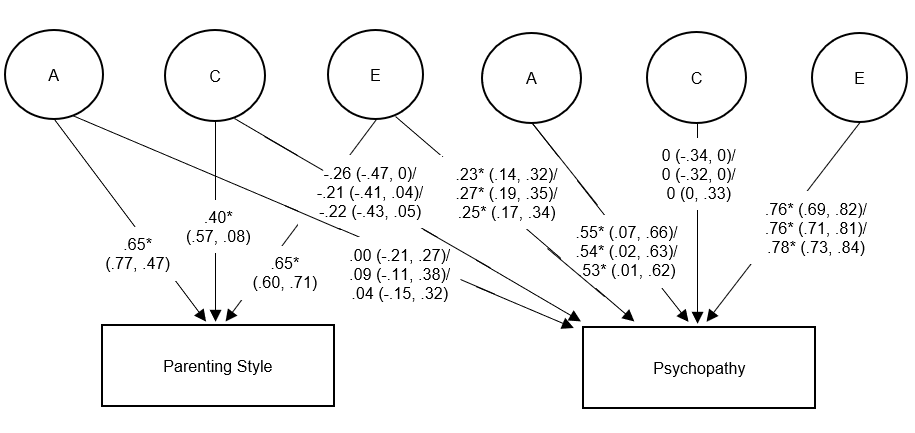


References

Brown, T. A. (2014). *Confirmatory factor analysis for applied research*: Guilford Publications.

Kenny, D. A. (2020). Measuring model fit.

Muthén, L. K., & Muthén, B. O. (2019). Mplus 8.3. Los Angeles, CA: Muthén & Muthén.

Neumann, C. S., Schmitt, D. S., Carter, R., Embley, I., & Hare, R. D. (2012). Psychopathic traits in females and males across the globe. *Behavioral Sciences & the Law, 30*(5), 557-574. doi:10.1002/bsl.2038

Paulhus, D. L., Neumann, C. S., & Hare, R. D. (2009). Manual for the self-report psychopathy scale. In: Toronto: Multi-health systems.

Yuan, K.-H., & Bentler, P. M. (2000). Three likelihood-based methods for mean and covariance structure analysis with nonnormal missing data. *Sociological methodology, 30*(1), 165-200.
